# Animal Welfare in Radiation Research: The Importance of Animal Monitoring System

**DOI:** 10.3390/vetsci10110651

**Published:** 2023-11-10

**Authors:** Monique Ribeiro de Lima, Daiani Cotrim de Paiva Campbell, Mariana Rietmann da Cunha-Madeira, Barbara Cristina Marcollino Bomfim, Jackline de Paula Ayres-Silva

**Affiliations:** 1 Center for Animal Experimentation, Oswaldo Cruz Institute, Oswaldo Cruz Foundation (FIOCRUZ), Rio de Janeiro 21041-250, Brazil; monique.lima@ioc.fiocruz.br (M.R.d.L.);; 2 Marcílio Dias Naval Hospital, Marinha do Brasil, Rio de Janeiro 20725-090, Brazil; 3 Laboratory of Experimental Medicine and Health, Oswaldo Cruz Institute, Oswaldo Cruz Foundation (FIOCRUZ), Rio de Janeiro 21041-250, Brazil

**Keywords:** radiation, hematology, animal endpoint, animal welfare, experimental animal models

## Abstract

**Simple Summary:**

In the years following World War II, scientists became very interested in the effects of radiation, especially after the atomic bombs were dropped on Japan. They started using animals like mice in their experiments to understand more about radiation. They wanted to know how much radiation is safe, how often to use it, how to care for people who suffer from this disease, as well as where to place cells during bone marrow transplants and how many cells to use. During these experiments, scientists noticed that sometimes the animals did not feel well or were in pain. So, they came up with ways to check on the animals’ health. They created scoring systems to assess the animals’ condition. This is important because we want to make sure animals are treated well in research. That is why scientists use these scoring systems to monitor the well-being of animals. Occasionally, different scientists use different rules, which can be confusing. For this reason, it is important to have common rules that everyone agrees on, which helps make sure that the research is accurate and that scientists from different places can work better together.

**Abstract:**

Long-term research into radiation exposure significantly expanded following World War II, driven by the increasing number of individuals falling ill after the detonation of two atomic bombs in Japan. Consequently, researchers intensified their efforts to investigate radiation’s effects using animal models and to study disease models that emerged post-catastrophe. As a result, several parameters have been established as essential in these models, encompassing radiation doses, regimens involving single or multiple irradiations, the injection site for transplantation, and the quantity of cells to be injected. Nonetheless, researchers have observed numerous side effects in irradiated animals, prompting the development of scoring systems to monitor these animals’ well-being. The aim of this review is to delve into the historical context of using animals in radiation research and explore the ethical considerations related to animal welfare, which has become an increasingly relevant topic in recent years. These concerns have prompted research groups to adopt measures aimed at reducing animal suffering. Consequently, for animal welfare, the implementation of a scoring system for clinical and behavioral monitoring is essential. This represents one of the primary challenges and hurdles in radiation studies. It is concluded that implementing standardized criteria across all institutions is aimed at ensuring result reproducibility and fostering collaboration within the scientific community.

## 1. Introduction

Animal welfare refers to the consideration of appropriate treatment for animal, encompassing their comfort, nutrition, and prevention of pain, fear, or distress. In addition, the animals must receive proper care, including their husbandry, physical and mental needs, and human treatment [1]. The Universities Federation for Animal Welfare (UFAW) is the oldest organization, founded in 1926, that works on animal welfare worldwide and concerns itself with promoting welfare in many fields of animal usage. The UFAW produced the first *Handbook on the Care and Management of Laboratory Animals* in 1947 [2]. In 1959, William Russell and Rex Burch introduced the enduring principles of humane welfare for experiment animals, which is until now known as the 3 Rs: reducing, refining, and replacing animal usage [3].

Radiation is the emission of waves or energy particles from an atom that can penetrate a solid medium, ionize molecules, or break chemical bonds, depending on the energy. Radiation can be divided into non-ionizing and ionizing and classified into alpha, beta, and gamma rays. Among these, gamma rays possess the smallest wavelength and the highest energy, leading to a host of side effects [4,5]. The infrared wavelength was discovered by William Herschel [6], whereas ultraviolet was found by Johann Wilhelm Ritter [7] at the beginning of the 18th century. Radiation has been very useful in medicine since the discovery of X-rays by Wilhelm Roentgen and his wife Bertha in 1895. Since then, radiation has been applied to diagnosis and treatment, such as radiotherapy, and research [4,5].

Radiation therapy, a critical medical application of radiation, comes in two main forms: external and internal beams. The external is the most often used and comes from photons, protons, or electrons. Photon machines are the most used and modern type that causes fewer side effects to the adjacent tissue. The most common type of external beam radiation is 3D radiation therapy, which uses images from computed tomography (CT), magnetic resonance imaging (MRI), and positron emission tomography (PET) scans to diagnose an affected area precisely. Other techniques in external radiation therapy include intensity-modulated radiation therapy (IMRT) and image-guided radiation therapy (IGRT) [8]. Internal radiation therapy could be solid or liquid, with the solid one, known as brachytherapy, being the most used. The main challenge in using radiation for human treatment is due to the damage of adjacent tissues, which would lead to too many cell deaths [9]. Patients typically experience complaints such as fatigue and hair loss, but other symptoms like nausea, throat problems primarily related to swallowing, and skin changes are very often side effects [10]. These symptoms are associated with acute radiation syndrome/sickness (ARS), which has been well-documented through protocols like the medical treatment protocols for radiation accident victims (METREPOL), which combine clinical symptoms/signs, and blood tests as prognostic indicators in different tissues [11,12].

Murine models, utilizing rodents like mice and rats, represent a crucial biological model applied to research involving ionizing radiation, stimulated after radiation accidents or terrorist attacks [13]. Radiation studies are also employed in bone marrow transplantation (BMT), graft-versus-host disease (GVHD), and graft-versus-leukemia (GVL) [14,15,16]. In addition, murine models can: 1—assess the efficacy of medical countermeasures; 2—identify and validate candidate biodosimetry assays for assessment of injury and radiation dose; 3—model acute radiation syndrome very well because they are small and allow perfectly total body irradiation (TBI) [11,13]. However, experiments involving ionizing radiation run into a diversity of side and systemic effects that lead to animal suffering, which led us to delineate the pitfalls and achievements of radiation research, with emphasis on bone marrow transplantation and the animal welfare associated with it. To this end, there must be a well-understood pathophysiological mechanism for radiation toxicity and the humane endpoint of animal study must be related to the desired benefit in humans. The humane endpoint of experimental studies is necessary to ensure animal welfare, and various intervention criteria have been used to minimize distress and pain [1]. In this sense, people have been trying to systematize and quantify subjective elements such as pain and distress to measure the accurate signals animals express [17,18,19,20,21,22].

This review aims to provide insight into the historical context of using animals in radiation research, with a specific focus on the establishment of murine models and the ethical considerations surrounding animal welfare in this field.

## 2. A Brief History of Radiation Research

The history of radiation research saw a significant turning point in 1895 when Wilhelm Conrad Roentgen and his wife Bertha discovered “X-rays” [23]. Soon after, in 1896, X-ray devices and radioactive materials were applied in physics, chemistry, and medicine. Since then, radiation has been used in many applications and studies, such as radiation therapy to improve the treatment of cancer patients [24]. Animal studies in radiation began around 1903 and showed that X-rays could produce cancer and destroy living tissues, with the skin, the blood-forming tissues, and the reproductive organs being the most vulnerable to radiation damage [25].

In the late 1940s, against the backdrop of the aftermath of World War II and the devastating atomic bombings in Hiroshima and Nagasaki, Japan, long-term research into radiation exposure experienced a significant surge [26]. The increased risk of cancer among the survivors of atomic bombs increased after 1952 with leukemia being the first to appear, and solid cancers starting ten years later, around 1961. Usually, leukemia cases appeared 2 to 25 years after radiation exposure, while solid tumors manifested 25 to 40 years to after [25,26]. Therefore, radiosensitivity research needs to be deepened in this context, and experimental research has increased.

Toward the end of the 1940s, many studies involving radiation research increased. One of the pioneers of experimental radiation and bone marrow transplantation was Jacobson et al. in 1949, who found that shielding the spleen of mice during lethal irradiation permitted their survival [27]. In the same period, Rekers et al. also studied the effects of radiation on animals. At this time, the challenge was to develop an experimental model for studying radiation in which the most expected result was the survival of the animals. They irradiated dogs with 3.5 Gy and closely examined the survival rate, along with the hematological and histopathological aspects of these animals [28]. Reker’s results showed that most of the animals died after irradiation. The mortality rate was higher still (80%) when they radiated and transplanted the whole bone marrow suspension directly into the femurs. However, when radiation was combined with bone and bone marrow transplantation into the femur, mortality decreased. Additionally, serial intravenous injections were associated with reduced morbidity and mild depression of hematological parameters. When marrows were transplanted into the right side of normal spleens without irradiation or when only spleens were irradiated with 25 Gy, none of the animals died. Conversely, when the whole animal was irradiated (3.5 Gy) and the marrow was transplanted into the spleen, the same mortality was observed in whole-body irradiated animals. Those experiments were essential to discover the importance of the definitive sites of proliferation of hematopoietic cells and provided some clues about how immune tolerance works. When the marrow was transplanted into the anterior chamber of the eye, the subcutaneous tissue of the dorsum of each ear, and the muscular and peritoneal surface of the abdomen, no successful transplantation of bone marrow was observed. They concluded that only intravenous injections of marrow transplants were effective in recovering the bone marrow, which was a significant advance [29]. Nonetheless, besides the discovery of MHC genes in 1936 by Peter Gorer [30], they still did not know about the histocompatibility theory, which resulted in the significant failure of the procedures, especially when the mice were not irradiated. Nevertheless, they yielded essential information about tolerance and compatibility [28,29].

In the 1950s, Jacobson et al. reported enhanced survival in mice subjected to whole-body irradiation when their spleens were externally shielded [31,32]. In these studies, protection was extended to one of the legs/femurs, a fraction of the liver, or the intestine. They observed increased survival, but less than when spleen protection had been performed [31], showing an important role played by the immune system, and which sites are essential for the proliferation and maintenance of hematopoietic cells.

Lorenz et al. took a significant step by lethally irradiating mice and guinea pigs, followed by the administration of hematopoietic cells from the same species (homologous) or from other species (heterologous). This intervention reversed the bone marrow collapse resulting from lethal irradiation doses, leading to increased survival [33,34]. Subsequently, Jacobson et al. hypothesized that radio-protected bone marrow and spleen cells could neutralize the toxins produced by radiation, make soluble factors that shielded them, or cause cellular seeding of the injected cells [31,35]. In addition, they understood the principles of histocompatibility, the existence of cytokines responsible for homing and attracting cells to the bone marrow, and the importance of the spleen during the homing process to the bone marrow.

Nowell et al. conducted experiments in which cells from alkaline phosphatase-positive rats were injected into mice that lacked alkaline phosphatase activity. In the case of heterologous transplantation, from 1 to 28 days they observed a growing detection of rat alkaline phosphatase-positive cells in irradiated mice. When the heterologous transplant was conducted into non-irradiated mice, the rat cells were detected until three days, after which only mice cells were observed [36]. Using those cells in this work was one of the first attempts to establish a detection method for transplanted cells. They also concluded that heterologous transplants experienced rejection after 30 days, despites the colonization of rat cells in the beginning, These findings advanced the concept of bone marrow transplant and immunity. Ford et al. intravenously injected spleen cells from young mice heterozygous for T6 translocation into irradiated fetal mice and identified them 5 to 49 days after injection [37]. This was another work establishing a cell marker to trace cells during the bone marrow transplant, which was a big concern then. Another improvement brought about by the group was the survival of animals for more than 30 days during the experiments.

In 1961, Till and McCulloch made a significant breakthrough by irradiating mice and transplanting bone marrow cells from other animals (donors) into the recipients a day later. This led to the emergence of myeloid and erythroid colonies in the spleen, which they termed “spleen colony-forming units” (CFU-S). Furthermore, the results showed that the number of colonies in the spleen was proportional to the number of bone marrow cells injected into the irradiated animals, suggesting that a specific part of the hematopoietic cell population could reconstitute hematopoiesis in vivo [38]. This was the first confirmation of this homing route for injected cells, in addition to the previous results obtained by Jacobson. Till and McCulloch also observed that injected hematopoietic cells could expand clonally in the spleen before homing to their definitive site, the bone marrow. In a subsequent study in 1963, the same group confirmed the clonal nature of bone marrow cells when they inherited a chromosomal alteration induced by irradiation. Upon transplantation into irradiated mice, all the cells in the spleen colonies exhibited the same radiation-induced alteration, affirming their descent from single cell and laying the foundation for the clonal theory of hematopoiesis, a widely accepted theory nowadays [39].

Since then, much research has been conducted to study the implications of irradiation of different organs, with bone marrow transplantation as one of the main targets of study. At the beginning of radiation research, the main concern was the damage caused by the radiation. As knowledge improved, the attention was focused on compatibility and how to treat the side effects of radiation. Another step was using radiation to treat neoplasms causing as few side effects as possible. The problems related to graft-versus-host disease and graft-versus-leukemia came together with using radiation in BMT. One of the great discoveries from this procedure was the beneficial effect of GVL, which comes from GVHD, which turned out to be an essential model for radiation in BMT research as it is a significant side effect in humans that increased the importance of experimental research [15,40].

Currently, BMT is a recognized treatment for a variety of diseases, including leukemia, myelomas, lymphomas, aplastic anemia, solid tumors, and others. In addition, other subjects, such as GVHD and the phenomenon of GVL, are essential for discovering key features in hematology, immunology, and radiation research [14,15,16]. Numerous animal models are used to research the mechanisms involving the effects of irradiation and BMT, which deal with key concepts on hematopoiesis, affecting the composition of bone marrow niches and the agents of tolerance to transplant rejection. Among the primary animal models employed in this research, rodents [33,34,41], dogs [28,29], lagomorphs [29], birds [42,43], and primates [44] stand out. These models have contributed significantly to the understanding of the effects of radiation.

## 3. The Importance and Pitfalls of Radiation Dose Rate, Animal Strains, and Injection Sites for Bone Marrow Hematopoietic Stem-Cell Transplant

Some factors have been studied and highlighted as necessary for establishing several disease models such as BMT, GVHD, and GVL studies: (a) the murine strain and its sensitivity to radiation, (b) the choice of a single or split dose, (c) the dose interval for each strain, (d) the time between irradiation and injection of hematopoietic cells, (e) the source and amount of hematopoietic cells injected, and (f) the site of injection of these cells [16].

The precise irradiation duration is determined by various factors, including radioisotope decay rates, the requisite extent of radiation exposure, and the specific type of ionizing energy source employed, such as X-rays or gamma rays (cesium or cobalt source, primarily) [16]. Another critical factor that must be observed for the success of BMT is the irradiation dose rate, which must be greater than 0.8 Gy/min for the depletion of receptor cells. Still, increased further to 1.3 Gy/min, the survival rate could diminish [45]. In our experience, we used to work with a single dose equal to or higher than 9.6 Gy with a six-hour interval between irradiation and injection of hematopoietic cells in the caudal vein of C57BL/6J mice.

The use of anesthetic before irradiation to minimize the distress as well as a subcutaneous injection of saline to minimize dehydration observed due to the pain and walking limitations after irradiation improved animal welfare. Pain was assessed by considering a combination of clinical signs with behavioral parameters that are indicative of pain. These clinical signs included moderate body hunched, eye closure (>50%), limited or no mobility, abdominal breathing, and lack of grooming. Using sedatives in the radiation protocol permitted a more humane injection of hematopoietic cells because the animals were less susceptible to stress. We also used opioid analgesics to minimize the pain after irradiation when clinical signs were observed such as body posture (hunched posture), general lack of grooming, eye appearance, and activity level.

The main challenges in radiation research persist in terms of reproducibility and comparability, as there is a significant disparity in the main parameters used in BMT models, such as the murine strain and dose employed, which could be single or fractionated, and the time between irradiation and injection of hematopoietic cells, which could range from to 1 to 24 h [46,47,48]. In addition, the number of hematopoietic cells injected could vary from 5 × 10^5^ to 7.5 × 10^6^, as well as the site of injection that could be retro-orbital, or intrafemoral, with the most used being the caudal vein [46,47,48,49,50].

For example, Cui et al. were among the first groups to investigate whether dose fractionation improves BMT outcomes. They found that fractionating the radiation into two equal doses of 6 Gy or 6.5 Gy, with a 4 h interval between irradiation and injection, resulted in lower mortality and improved engraftment of the grafted bone marrow compared to a single dose regimen, using C57BL/6 and C3H/HeN mice [14]. In addition, dose fractioning has allowed higher irradiation doses (8–10 Gy), increasing the survival of animals, and humans, and decreasing the chance of relapses [51].

Radiation sensitivity may explain the results observed in human BMT and the differences obtained among murine strains. Wide variations along with animal models concerning species, strain, dose, dosage rate, time, endpoint, level of supportive care, and protocols make it difficult to standardize and validate these models for studies of radiation exposure and prophylactic measures and treatment. Therefore, in building a consensus regarding the best animal models, assessment tools and endpoints should be considered. Other genetically determined differences between mouse strains may affect tissue responses after irradiation. These discoveries are essential so that an irradiation protocol and therapeutic possibility can be applied to humans.

Among the most commonly used strains in animal experimentation, C57Bl/6 (10–11 Gy) is more tolerant to radiation than BALB/C (9 Gy) and CBA/J (8 Gy) [14,16,52,53], and the strains derived from heterologous crosses are even more resistant, such as the B6D2F1 (C57BL/6J × DBA/2), which can tolerate 15 Gy of total body irradiation (TBI) [15]. C3H/HeN is not very sensitive to single-dose radiation. However, it becomes susceptible when irradiated with low fractional dose conditioning [14]. Other strains such as 129/J [52,53] are sensitive to seasonal variations, being less resistant to summer radiation [52]. To summarize the relationship between mice strains and their tolerated dose, we could indicate the studies by Roderick, [52], Yuhas and Storer [53], and Reddy et al. [15].

## 4. Monitoring System for a Humane Endpoint in Radiation Research

The humane endpoint can be defined as the earliest indicator of pain and/or discomfort in an experimental animal. It is established based on ethical and scientific guidelines to limit or end pain and discomfort through specific actions [18]. Consideration and appropriate use of these actions can ensure optimal welfare for animals used in irradiation-related research. For this purpose, over the years, systems were developed to determine individual clinical status, discomfort, and any signs different from average to predict early intervention to not let the animals go through until imminent undesired death.

In humans, there is a guide called “*Medical Treatment Protocols for Radiation Accident Victims—METREPOL*”, which combines clinical symptoms/signs and blood tests as prognostic indicators of acute radiation syndrome/sickness (ARS) in different tissues [11,12]. However, rodents, especially murids, serve as the primary and best-studied model in experimental animals. Therefore, a scoring system for clinical signs as well as biomarkers of severity has been created to screen them [11,22,44,54].

ARS, or radiation toxicity, is characterized by progressive multiorgan dysfunction (two or more organ systems) after exposure to ionizing radiation, which mainly affects the hematopoietic, gastrointestinal, and neurovascular systems and, to a lesser extent, can also cause idiopathic interstitial pneumonia and late cataracts [14,21,22,51]. The severity of ARS is determined by factors such as the overall dose, dose rate, radiation quality, and the proportion of the irradiated body. Four clinical stages characterize its progression: prodrome, latency, illness, and recovery or death [20]. An increase in radiation dose could cause death in mice due to the destruction of the bone marrow, sloughing of the mucous layer of the gastrointestinal system, skin burns, and collapse of the neurological and cardiovascular systems [20]. LD50/30, the dose at which fifty percent of animals will die within thirty days, is one of the classic ways that experimental animals have been measured during radiation experiments, as well as a survival curve plot where the exploratory endpoint to determine the effectiveness of the dose in question is the death and moribund state of mice. Due to these lesions and extreme animal suffering, a scoring system, and the degree of severity of the radioactive lesions were created in human and in animal models.

Cooke et al. (1996) proposed one of the first animal monitoring systems when studying idiopathic pneumonia syndrome induced by acute GVHD [21]. Based on studies of murine models of irradiation, a murine intervention scoring system (mouse intervention scoring system—MISS) was created by the Veterinary Sciences Department (VSD) of the American Armed Forces Radiobiology Research Institute (AFRRI), which aims to identify early signs of animal suffering following irradiation [22].

A severity scoring system comprising several parameters facilitates the identification of animals that require enhanced monitoring after irradiation [11]. Multiple monitoring systems have been suggested since then, including stress, cancer, and behavioral monitoring of murids after irradiation, an experience gained after initial radiation studies [17,18,19]. However, Nunamaker et al. enhanced existing tables for monitoring irradiated animals to minimize the stress associated with ARS, resulting in the first cageside observational scoring system [20].

The mouse intervention scoring system (MISS) is an individual monitoring system designed to enable earlier and more efficient intervention measures. It has undergone multiple improvements and adaptations after rigorous staff training to maintain consistency and is now in its third version (MISS3). This monitoring system evaluates clinical signs and establishes criteria for prognosis and outcome [22]. Clinical score parameters observed in animals include hunching, hair grooming, weight loss, diarrhea, animal appearance (skin, eyes), breathing-related features, general and provoked behavior (mobility, ataxia), and clinical signs, such as changes in weight and temperature [22]. In our experience, signs of dehydration, pain, and weight loss were evident within the first eight days. From day 08 to 14, we monitored signs of weight loss, body temperature variation, activity, and general health. During this monitoring period, survival was more likely if the animals did not exhibit these signs. Pain control and veterinary care during animal monitoring are essential for the survival of the animals and a limiting condition for animal recovery. Promoting their welfare contributes to the success of the research. On the other hand, death is a very likely outcome whether their weight and temperature drop by more than 20% and 35.2 °C, respectively [16]. In summary, Table 1 presents the main authors and their proposed animal monitoring systems, study models, animal monitoring parameters described in the literature, and their advantages and disadvantages.

Among the various criteria analyzed, it is essential to emphasize that several studies have drawn attention to daily weight monitoring, with fluctuations even more significant than 20% of initial body weight [16,20,22]. The post-irradiation weight oscillation is due to dehydration and is responsible for approximately 10% of the weight. Therefore, water and food be offered to animals close to the ground during the first 10 days after irradiation because of weakness and locomotion difficulty. Food should be softened, preferably in pasty format, autoclaved, and have rigorous microbiological control. Water should be acidified and contain antibiotics to minimize contamination by bacterial infectious agents. One option is to offer water in a gelled form [16]. In our experience, we supplemented the pre-irradiation diet with autoclaved dried fruits and oil seeds, which was very important for maintaining the weight of the animals, especially in the 24 h following the procedure [63,64,65]. In addition, hypercaloric autoclaved pasty food was offered after irradiation to recover convalescent animals. Pre-and post-irradiation veterinary care was essential for the survival of the animals during the studies.

However, in studies involving radiation and models of solid neoplasms, weight may not be the ideal criterion for monitoring animals, as tumor growth and loss of muscle mass and dehydration can mask weight fluctuations resulting from increased tumor mass [19]. For these cases, a comparison between muscle mass and body fat, the body condition score, should be an alternative monitoring method, even in experiments with irradiation [16,66].

Histopathological criteria can be essential analysis tools. However, they do not allow animal monitoring throughout the experiment, as they require the euthanasia of the animal for studying the organ after the investigation [49]. Thus, other monitoring systems that allow in vivo follow-up are being used in addition to traditional clinical/behavioral criteria. The most modern experimental endpoint criteria are the use of non-invasive images, which have the potential to assess complex biological processes with a large amount of information, with excel refinement.

Some methods employed to track hematopoietic reconstitution using noninvasive images allow post-transplant engraftment. Different modalities can be used to screen the engraftment of hematopoietic stem cells transplantation, such as bioluminescence imaging (BLI) [67,68], fluorescence imaging (FLI) [69], positron emission tomography (PET) [70], single photon emission computed tomography (SPECT) [71], and magnetic resonance imaging (MRI) [72]. Some methodologies have advantages over others, with BLI, FLI, and PET being relatively less expensive, in contrast to poorer spatial resolution owing to the limited depth of light penetration into tissue compared to SPECT and MRI. However, combining both techniques enable the combination of anatomical and molecular information [73]. Moreover, it is important to consider the quantitative analysis of the graft and its functionality when evaluating its efficiency, an aspect that has yet not to be addressed in most in vivo imaging studies of BMT [50].

## 5. Discussion

The development of a monitoring system should be geared towards an individual animal and the level of pain or discomfort experienced by each animal needs to be reported. It is important to note that using of objective measures to assess the level of pain or distress experienced by the animal during the procedure should capture any welfare issues (expected and unexpected) that occur during the experiment [18].

More modern and effective clinical monitoring techniques can improve numerical scoring systems that classify clinical–behavioral signs according to their displeasure [20]. An ideal assessment system should include simple and objective appraisals that can be applied consistently to detect the onset of pain and monitor its development. Experience, well-trained staff, and professional judgment will allow for better objective scoring. As such, score systems must be carefully constructed in discussion with all those involved in the animals’ care and re-evaluated and modified based on observations made during a pilot study. In this way, it will be possible to identify actions that may refine the procedure or establish early outcomes and will allow for rapid veterinary intervention [16].

Monitoring systems can use binary cardinal signals (present or absent), simple categorization (absent, moderate, apparent) or they can be scaled by assigning more weight to the discomfort signs on a scale aggravation ranging from normal to the highest. The use of scores for variables and different criteria can improve the accuracy of a clinical-behavioral assessment in animals. By assigning a score to each standard, it is possible to combine various data to produce a summary measure more representative of the general animal state or the severity of its condition. It is crucial to ensure that each criterion is weighted appropriately against the others and that the score combination provides a valid and reliable measure. Furthermore, it is essential to consider the clinical relevance of each criterion and how they relate to the experimental protocol. In this way, the total score obtained indicates how far the animal deviates from normal at the time of evaluation. Consequently, the score should prompt action, such as medical treatment, to prevent the animal from deteriorating further.

In conclusion, a single evaluation system has yet to be made available, due to the wide variation in behavior and responses among different species, strains, and individuals, in addition to the specificities of each experimental procedure. In general, when systems are more straightforward, they require less training and are more reproducible, in contrast to the more complex ones, which tend to be more specific. Most improvements have been made in animal care, as the immune compromise of mice persists for a period after BMT. Therefore, animals must be in systems that maintain their sanitary condition, without risk of cross-contamination. Veterinary care measures are essential and can determine the success of experimental protocols, such as preventive antibiotic therapy, and to provide care for food and water quality.

The routes of intravenous administration and serial blood sampling in mice are limitations that require attention, especially when searching for solutions that promote animal welfare. Thus, inoculation routes and blood collection in different anatomical sites (e.g., gingival and sublingual vein) should be preconized to minimize the potential damage that may be caused to the mice during the development of the experiment [74,75,76].

The use of fractionating radiation doses should be tested to refine the technique to reduce the morbidity and mortality of experimental animals [51]. In addition, a better understanding of side effects in the fractionation system could be important to improving veterinary care and the predictive value of death.

The use of supportive care must be carefully considered to assist the animal’s basic needs so that it can fully respond to the irradiation protocol and BMT. Examples include providing comfort with an environment enriched with supplemental heat and temperature regulation (e.g., nesting material), parenteral fluids, and nutritional support, such as high-quality nutritional sources that are easy to access and digest. The potential effect of supportive care should not be underestimated, and its possible influence on experimental results should be critically evaluated, just as in pharmacological interventions [16].

The parameters used for the experimental endpoints should be monitored, recorded throughout the research, revised as needed, and included in the methodology and results sections of the publications. In addition, all staff should receive appropriate training and competency assessments to monitor signs of adverse effects. In vivo longitudinal imaging can be an integral part of the methodology refinement to improve mouse models of irradiation, BMT, and other diseases [16].

A better understanding of these murine models is the key to establishing more effective irradiation protocols in treating several oncohematological diseases and bone marrow transplants. For example, TBI protocols in BMT conditioning for aplastic anemia and acute lymphoblastic leukemia, as a better way to achieve GVL and diminish the chance of GVHD. On this matter, it is also essential to consider the specific factors that influence remission after BMT such as donor–recipient infections such as cytomegalovirus (CMV) serologies, social status, virus reactivations, and donor–recipient differences in weight, among others, which reflect animal care in a germ-free environment.

## 6. Conclusions

It is essential that preclinical models in irradiation and BMT research accurately reflect modern clinical practice, both in choosing the optimal animal model and in concise radiation exposure conditions. In addition, the clinical–behavioral monitoring of animals and the use of in vivo imaging techniques provide an excellent opportunity to improve animal welfare and refine processes, maximizing the potential of research and its reproducibility.

Therefore, using different methodologies to predict animal death and avoid unnecessary pain and discomfort is essential. Adopting additional criteria for the experimental endpoint decreased the variability between studies and reduced the observational bias. Finally, more correlation studies between clinical scores and pathophysiological assessments also need to be carried out to increase the accuracy of the death predictive value, avoiding unnecessary pain and discomfort. Establishing standard endpoint criteria among institutions through prospective comparison of the systems to determine the best one to be employed in each protocol will improve the reproducibility of the results, and strength the entire scientific community, which is a great challenge in irradiation studies.

## Figures and Tables

**Table 1:** Features analyzed in mice models by several authors for building experimental endpoint tables for animal monitoring. Adapted from Naserian et al. [49]. Legend: GVHD—graft-versus-host disease; TBI—total body irradiation.

Authors	Year	Model	Features Analyzed	Advantages	Disadvantages
Cooke et al. [21]	1996	GVHD	Weight loss, posture, activity, fur texture, skin integrity	Quantitative and semi-quantitative sheets with simple categories. Less complex scoring system.	Non-specific clinical signs. Overestimate or underestimate pain and distress experienced by animals in some situations.
Lloyd & Wolfensohn [18]	1999	Emphysema and encephalomyelitis	Appearance, food and water intake, clinical (respiratory) signs, natural behavior, provoked behavior	Combination of signs can be used to indicate overall severity of the procedure. Inclusion of specific parameters for the disease model (respiratory signs), increasing the sensitivity and specificity of the humane endpoint.	Validation of observation sheet. Variability between observers.
Morton [17]	2000	Diabetes, vaccine	Weight loss, posture, activity, fur texture, skin integrity, general appearance, food and water intake, type of breathing, behavioral assessments, diarrhea, dehydration, eye appearance, social isolation, temperature, pinched face, not grooming, vocalization, distended abdomen/swollen, blood sugar level, polyuria.	Quantitative and semi-quantitative sheets with Simple categories. Less complex scoring system. Single signs or combination of signs can be used to indicate overall severity of the procedure. Score sheets reveal patterns of recovery or deterioration of health status and provides information on the effect of procedures on animals	Establish and limit cut-off points for clinical intervention and humane endpoint.
Anderson et al. [55]	2003	GVHD	Skin ulcers with different size of alopecia and the site of skin lesion	Objective scoring and specific parameters for skin analyze.	Establish and limit cut-off points for clinical intervention and humane endpoint. Validation of observation sheet.
Mutis et al. [56]	2006	XenoGVHD	Weight loss, mobility, and general appearance	Quantitative and semi-quantitative sheets with simple categories. Less complex scoring system.	Non-specific clinical signs. Overestimate or underestimate pain and distress experienced by animals in some situations.
Paster et al. [19]	2009	Cancer	Body weight, appearance, behavioral assessments to evaluate morbidity (natural and provoked), body condition scoring	Combined assessments more accurately illustrate animal health status.	Behavioral signs may overlap with clinical signs. Variability between observers.
Wilson et al. [57]	2009	GVHD	Weight loss, posture, activity, fur texture, skin integrity, and diarrhea	Quantitative and semi-quantitative sheets with simple categories. Less complex scoring system.	Non-specific clinical signs. Overestimate or underestimate pain and distress experienced by animals in some situations
Castor et al. [58]	2010	GVHD	Weight loss, posture, activity, fur texture, skin integrity, diarrhea, and occult blood in feces	Quantitative and semi-quantitative sheets with simple categories. Less complex scoring system.	Non-specific clinical signs. Overestimate or underestimate pain and distress experienced by animals in some situations
Lai et al. [59]	2012	GVHD	Weight loss, posture, activity, fur texture, skin integrity, and diarrhea	Quantitative and semi-quantitative sheets with simple categories. Less complex scoring system.	Non-specific clinical signs. Overestimate or underestimate pain and distress experienced by animals in some situations
Nunamaker et al. [20]	2013	TBI	Body posture, eye appearance, activity level	Combined assessments more accurately illustrate animal health status.	Variability between observers.
Budde et al. [60]	2014	GVHD	Posture, activity, fur/skin, and diarrhea	Quantitative and semi-quantitative sheets with simple categories. Less complex scoring system.	Non-specific clinical signs. Overestimate or underestimate pain and distress experienced by animals in some situations
Doisne et al. [61]	2015	GVHD	Weight loss, posture, activity, and fur texture	Quantitative and semi-quantitative sheets with simple categories. Less complex scoring system.	Non-specific clinical signs. Overestimate or underestimate pain and distress experienced by animals in some situations
Koch [22]	2016	TBI	Appearance, respiratory rate, general behavior, provoked behavior, weight loss	Promotes a more careful observation of the animals by all those involved in the critical moments of the experiment. Single signs or combination of signs can be used to indicate overall severity of the procedure.	Validation of observation sheet. Variability between observers.
Naserian et al. [49]	2018	TBI	Weight loss, hunched posture, fur texture, skin integrity, and diarrhea	Less complex model binary scoring and reproducible system.	Less specificity and variability between observers.
Kumar et al. [62]	2022	TBI	Inability of the mouse to right itself, limb paralysis, abdominal breathing, a constant twitching, trembling, or tremor that lasted for more than 10 s, or greater than 35% weight loss	Single signs or combination of signs can be used to indicate overall severity of the procedure.	Qualified technical team with knowledge in biology, ethology, and animal physiology Variability between observers.

## Data Availability

No new data was created for this paper.
